# Arthroscopic utilization of ChondroFiller gel for the treatment of hip articular cartilage defects: a cohort study with 12- to 60-month follow-up

**DOI:** 10.1093/jhps/hnab002

**Published:** 2021-07-31

**Authors:** Jacek Mazek, Maciej Gnatowski, Antonio Porthos Salas, John M O’Donnell, Marcin Domżalski, Jakub Radzimowski

**Affiliations:** 1 Jan Kochanowski University in Kielce, Orthopaedic and Traumatology Clinic, Kielce, Poland; 2 Center for Specialized Surgery Ortopedika, 03-152 Warsaw, Poland; 3 Hip Arthroscopy Mexico, Av. Frida Kahlo 180, Monterrey, Mexico; 4 Hip Arthroscopy Australia, 21 Erin Street, Richmond, Victoria 3121, Australia; 5 Orthopedic and Trauma Department, Medical University of Lodz, Lodz, Poland; 6 Orthopaedic and Traumatology Clinic WUM, Miedzyleski Hospital Warsaw, Warsaw, Poland

## Abstract

ChondroFiller gel is an absorbable collagen implant. It serves as a protective cover for the cartilage defects, allowing chondrocyte migration into the lesion. The implant consists of collagen (Type I) and is derived from veterinary monitored rats. This study evaluates the use of ChondroFiller gel in the treatment of cartilage lesions during hip joint arthroscopy. A prospective study was conducted on a group of 26 adult patients. All patients had an existing femoroacetabular impingement together with acetabular cartilage lesions >2 cm^2^. All patients underwent hip arthroscopic surgery and the lesions were treated using ChondroFiller gel. The cartilage tissue healing was evaluated postoperatively using MRI. A total of 26 patients, including 5 females and 21 males, all with articular cartilage lesions, were included in the study. Cartilage healing conditions were evaluated for all patients, and the difference between pre- and post-surgery conditions was statistically significant. The follow-up scores have been acquired from 21 out of initial 26 patients (2 were disqualified after receiving THR, 3 could not be reached by researchers) after 3, 4 and 5 years consecutively with 17/21 patients having good/excellent results. The use of ChondroFiller gel during arthroscopy of the hip for acetabular cartilage lesions is an effective treatment technique. Encouraging long-term results have been observed, but further research on larger group of patient is required to better assess the full value of this technique. Patients with pre-existing osteoarthritis (Tönnis 2–3) have poor results.

## INTRODUCTION

The articular cartilage is a very particular connective tissue in the joints that has a specific function. This tissue principally acts as a shock absorber and allows for the motion movements in the joints by facilitating the transmission of loads with provision of a smooth, congruent and lubricated surface. Articular cartilage has no blood vessels and nervous tissue (lymphatics and nerves), i.e. it is avascular and aneural. Its avascular environment makes it dependent on diffusion through its matrix for nutrition. The diffusion of nutrients from subchondral bone vessels and synovial tissue plays a critical role in maintaining normal adult cartilage homeostasis and function. It has long been recognized that partial and full-thickness lesions in the cartilage have limited capacity for repair, especially if the injury is followed by necrosis. In this context, the health of the articular cartilage and its preservation is of vital importance [1]. The two main reasons that limit the cartilage regeneration include (i) the avascularized nature of cartilage tissue, and (ii) chondrocytes have limited synthetic and mitotic potential. Despite the development of several innovative procedures in the areas of cell therapy and tissue engineering, the treatment of cartilage defects remains a significant challenge in our daily practice. Previously, to avoid continued pain and disability, joint arthroplasty was considered as the best option of treatment for deep chondral lesions [2–4]. A cartilage regenerative therapy would be greatly preferable, which will not only be long-lasting for the cartilage health and joint functioning but also supersede other therapeutic techniques as being less traumatic and complicated for the patients. Some reports suggest the use of bio-adhesives for the treatment of delaminating articular cartilage lesions, which has shown some encouraging results [5]. However, the method that is being used most often to treat cartilage lesions are microfractures, followed by cell-free repair approaches [6]. In this context, the gel implant, ChondroFiller (Meidrix Biomedical, GmbH) used during arthroscopy, has been suggested to be a promising alternative. The advantage over other cartilage treatment procedures, e.g. microfracture surgery [7–9] together with osteochondral transplant (OCT) surgery or autologous chondrocyte transplantation (ACT) [10] because it is used without the need to perform microfractures which might prove difficult to perform in the hip joint and without the need of a lengthy time cell culture [11]. The ChondroFiller is a stable collagen hydro solution composed of the acellular matrix that possesses the consistency of a solid gel that is intended to serve as a protective cover of the acetabular cartilage lesions allowing chondrocyte migration into the defect. The implant consists of collagen (Type I) solution and is derived from veterinary monitored rats. Once the ChondroFiller gel implant is inserted into the defect, the migration of stem cells and cartilage from the surrounding tissue begins into the collagen matrix, which in return stimulates the healing of the cartilage. The purpose of our study was to evaluate the usage of ChondroFiller gel in the treatment of cartilage lesions in the hip joint during arthroscopy by using clinical and non-invasive MRI assessments.

## METHODS

This is a prospective study of patients presenting with acetabular cartilage lesions bigger than 2 cm^2^ at the time of hip arthroscopy due to femoroacetabular impingement. In patients, severe chondral damage (3 and 4 ICRS) was found during the hip joint arthroscopic examination. Exclusion criteria included inflammatory diseases, kissing acetabular lesions, lesions smaller than the 2 cm^2^, Tönnis grade three lesions, avascular necrosis (AVN) and developmental dysplasia (DDH). The study was performed with institutional review board (IRB) approval from Ortopedika private hospital in Warsaw, Poland.

All patients gave written informed consent for the surgery, clinical tests and MRI follow-up before participation.

### Surgical procedure

**Fig. 1.**
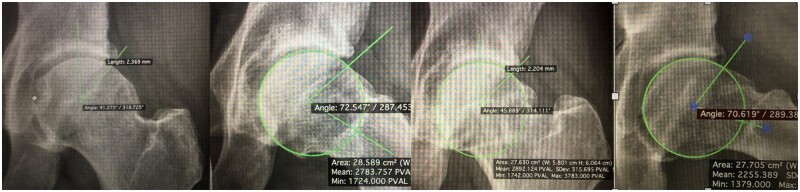
X-Ray of a patient who was treated using ChondroFiller gel (AP and lateral view).

Hip arthroscopy was performed in the modified supine position, with traction used to distract the joint under fluoroscopic guidance. Standard 70° arthroscope was used during the surgery. Entry was through the anterolateral (AL) portal, followed by the mid anterior (MAP) portal and through the (DALA; distal anterolateral) portal, to improve visualization. Capsulotomy was performed between AL and MAP. All Patients underwent rim recession and acetabuloplasty to remove the acetabular deformity (26 cases), and the recession of the femoral deformity (26 cases; Fig. 1). In three cases, partial labral resection was carried out; six cases had labral reconstruction and 17 cases labral repair.

**Fig. 2.**
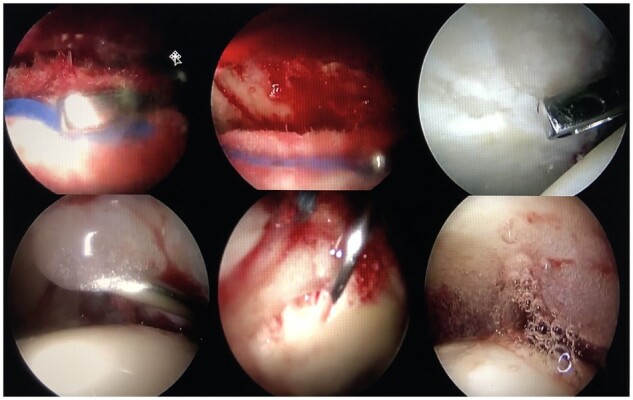
In the top line, acetabular chondral defect prepared for ChondroFiller introduction with the saline solution removed, and the defect cleaned with a swab. ChondroFiller filling acetabular chondral defect using a spinal needle is shown at the bottom line.

Two sutures were introduced through the capsule before the insertion of the ChondroFiller, so it would easily close at the end of surgery. ChondroFiller was then inserted after the saline solution was sucked from the hip joint, and a swab was used to dry the chondral defect. To make the joint drier, when sucking out the saline solution, the faucet of the arthroscopic mantle was kept opened to pump air into the joint; this manoeuvre improved the quality and vision of the defect to introduce and properly place the ChondroFiller gel. Spinal needle was used for implantation (Fig. 2). After ChondroFiller placement, hip traction was maintained for 12 min to initially harden the ChondroFiller. After that time the hip traction was slowly released and two previously prepared sutures closed the capsule.

### Post-operative rehabilitation regimen

Patients had no weight bearing following surgery on the operated hip for 3 weeks, partial weight bearing was allowed after that period. Passive range of motion began the first-day post-operative with a CPM machine that had fixed angle range from 0° to 30°, hip abduction and external rotation was allowed up to 20° for 4 weeks. During Week 5, the flexion of the hip was increased to 90°. Full weight-bearing and full range of motion were allowed after 6 weeks post-op [12].

### Outcome measures

All the relevant data was collected postoperatively with specified time intervals from 6 months until 60 months. Hip osteoarthritis was graded using the Tönnis classification. The hip function was assessed pre- and post-operatively using the modified Harris Hip Score (mHHS). MRI was performed after six months of the treatment to assess cartilage healing. A total of 21 patients were assessed using mHHS scale in the period ranging from 3 to 5 years after the surgery. Three patients were assessed after 3 years post-op, 15 patients 4 years after the surgery and three patients after 5 years.

### Statistical analysis

Tönnis classification system, Wilcoxon matched-pairs signed-ranks test and modified Harris Hip Score analyses were utilized to determine the difference between patient groups and their conditions both pre- and post-operative status, where applicable.

## RESULTS

**Fig. 3.**
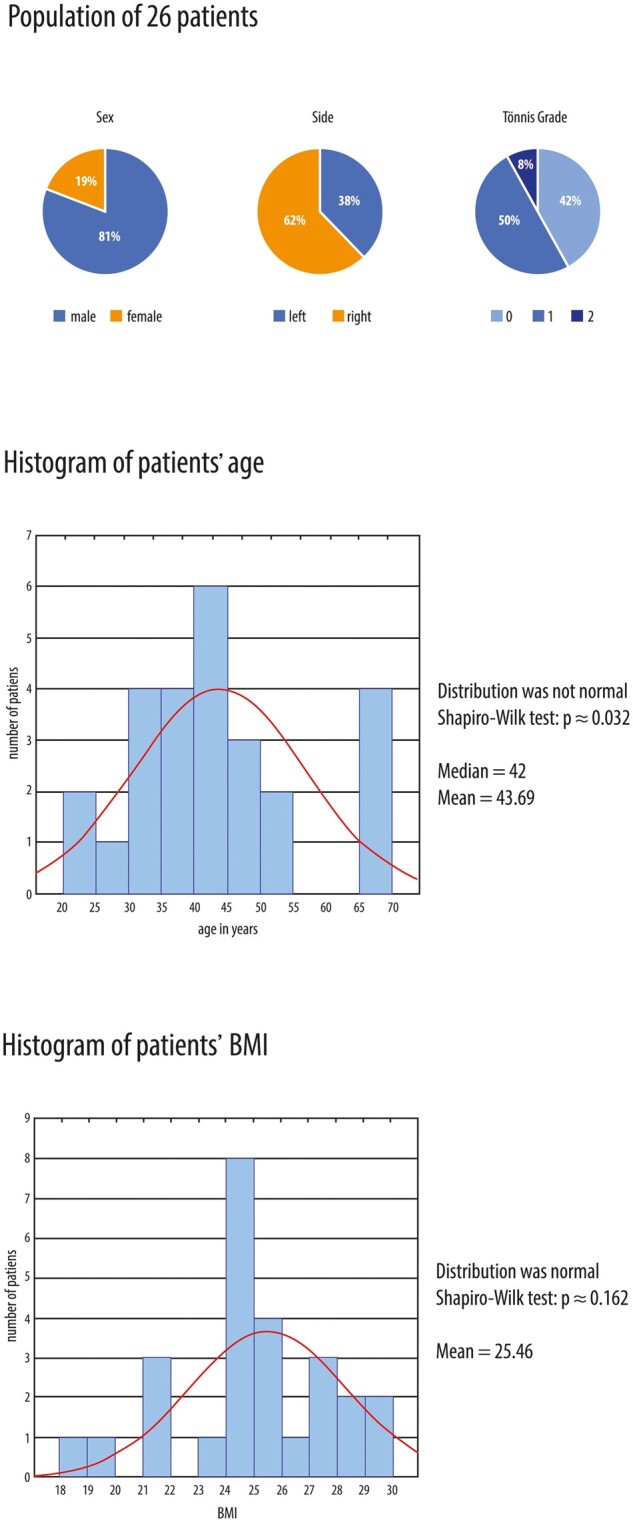
Statistical analyses and data showing (**a**) Patients' gender, hip side and Tonnis grades, (**b**) patients' age and (**c**) patients' BMI.

From 2015 to 2017, 26 hips with acetabular cartilage defects were treated using ChondroFiller gel. This surgical technique was used in patients presenting with femoroacetabular impingement syndrome, and all cartilage lesions were >2 cm^2^. Cartilage lesions were found and treated in all cases with changes in the second and third acetabular zones. The study group consisted of 19% females and 81% male patients. The mean age of this reported cohort was 42 years, with a median of 43.69 years (range 20–70 years, Shapiro–Wilk *P*-values ≈ 0.032). The body mass index (BMI) was in the range of 18–30 with a median value of 25.46 and Shapiro–Wilk *P*-values ≈ 0.162, indicating a normal distribution. Of the whole group of patients, 62% were having a problem on their left side while 38% were with right side hip cartilage lesions. Based on Tönnis classification, 42% (*n* = 11) of patients were Grade 0 indicating mild signs of lesions, 50% (*n* = 13) were included in Tönnis Grade 1 with moderate damage, while 8% of patients (*n* = 2) reported with Tönnis Grade 2 (Fig. 3).

The median improvement in postoperative mHHS was 33 (range 4–37). Results of all the patients Grade 0 and Grade 1 in Tönnis classification were rated as good (4) or excellent (20). The only two poor results in mHHS (both scored 68) were found in patients whose preoperative radiograms had been graded as Tönnis-2. The overall improvement was found to be statistically significant (Wilcoxon Matched Pairs Test, *P* < 0.0001). No correlation could be found with age within each grade. Also, data showed no correlation of score with gender and/or BMI. These results are comparable with previous data reported [7, 13].

**Fig. 4.**
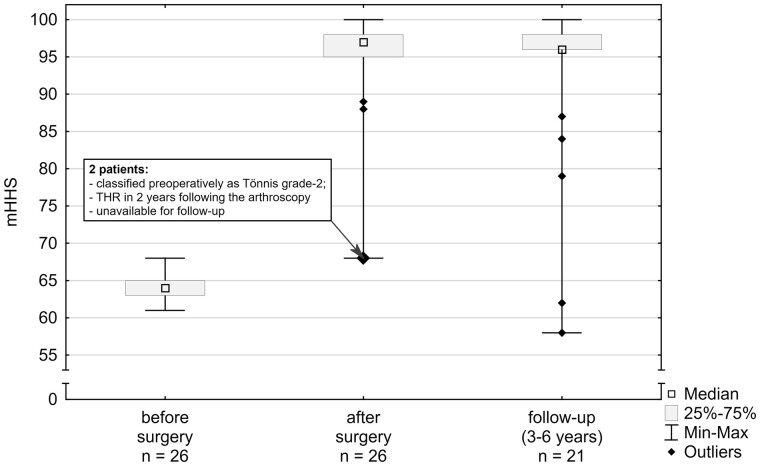
Box–Whiskers plot of modified Harris Hip Score. Median score of improvement after the surgery was 33 (min 4, max 37).

The follow-up scores have been acquired from 21 patients of initial group of 26 (two were disqualified after receiving THR, three could not be reached by researchers) between Year 3 and 6 after the surgery. Majority of the patients maintained good or excellent results (17/21, 81%) There was no statistically significant difference between follow-up and post-operative results [7, 13] (Fig. 4).

**Fig. 5.**
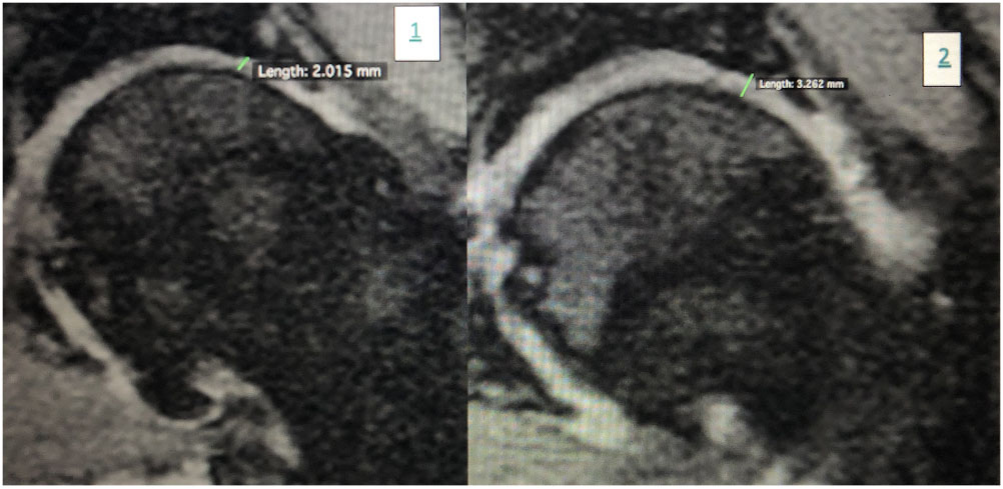
MRI (Siemens 3,0 T—t1_tirm_cor) of a 31 years old man. (1) Pre-operative image, with the chondral defect and chondrolysis in the acetabulum visible. Joint space 2 mm (2) MRI image of the same patient 1-year post-operatively with ChondroFiller gel. Joint space 3.2 mm.

In our study group of 26 patients, acetabular cartilage defects were treated with ChondroFiller gel implant. MRI control examinations were done regularly for all patients. MRI was performed to evaluate all hips post-operatively. A 3-Tesla MRI scanner (Siemens) was used. Cartilage defects treated with ChondroFiller gel in 24 patients in the group had significant improvement in healing results after 6 months and 1 year (Fig. 5).

## DISCUSSION

The biological response and restrictions in cartilage defect repair exist within joints after injury, and because of that many surgical techniques in tissue engineering have evolved using cells, 3D scaffolds and bioactive factors [14–19]. Several studies have addressed the cell-free treatment of cartilage defects and have been used as control groups for a cell-based approach in a comparative manner [20, 21]. In our study, satisfactory results were obtained with an excellent affirmation of defect healing conditions. The 27-point difference in the score improvement among Tönnis 0 and 1 was statistically significant and was more than above the 8.3 value [22], which is the minimal clinically significant difference to evaluate improvement after hip arthroscopy. In terms of the mHHS the outcomes are lower for those two patients with Tönnis 2. Although the mHHS improved to 64 and 68, both patients were considered poor outcomes. This is consistent with the findings of Byrd*et al*., who suggested that evidence from their study supports the view that joint space <2 mm and high Tönnis grade changes are indicators of unsatisfactory results in hip arthroscopy and represent objective contraindications [23]. However, it is the perspective of the authors that Tönnis classification for osteoarthritis acts as a primary indicator of the extent of pathology and lesions. MRI evaluation of the defects in the present study showed that in the majority of the cases, i.e. >90%, there was significant healing of the defect, during observation postoperatively, at 6 and 18 months, respectively, as evidenced by filling of the cartilage defects. It is accepted that cartilage regeneration and tissue remodelling generally take years after implantation, and cartilage remodelling can be visible on MRI [24]. Therefore, it will be essential to obtain results and studies with a longer follow-up.

## CONCLUSION

Hip arthroscopy and preservation surgery of acetabular cartilage lesions with the aid of ChondroFiller gel results in significant improvement at a minimum follow-up of 1.5 years. Among the findings, the best short-term outcomes are observed with a short period of pre-operative symptoms and good fill grade. The results suggest that the use of ChondroFiller gel for arthroscopic repair for acetabular cartilage lesions is effective, especially in defects larger than 2 cm^2^ in patients with Tonnis <2. Some encouraging long-term results have been observed, but further research on larger group of patients is required to better assess the full value of this technique. In our opinion, patients with Tonnis 2 and 3 are not good candidates for this procedure.

## CONFLICT OF INTEREST

No author has any conflict of interest related to this work.

## DATA AVAILABILITY STATEMENT

All relevant data are within the paper and its Supporting Information files.

## LEVEL IV EVIDENCE STUDY

 
